# Determination of Breast Metabolic Phenotypes and Their Associations With Immunotherapy and Drug-Targeted Therapy: Analysis of Single-Cell and Bulk Sequences

**DOI:** 10.3389/fcell.2022.829029

**Published:** 2022-02-14

**Authors:** Ming Bai, Chen Sun

**Affiliations:** ^1^ Second Department of Medical Oncology, The First Hospital of China Medical University, Shenyang, China; ^2^ Department of Radiology, Shengjing Hospital of China Medical University, Shenyang, China

**Keywords:** breast cancer, metabolism phenotype, non-negative matrix factorization, immunotherapy, drug-targeted therapy

## Abstract

Breast cancer is highly prevalent and fatal worldwide. Currently, breast cancer classification is based on the presence of estrogen, progesterone, and human epidermal growth factor 2. Because cancer and metabolism are closely related, we established a breast cancer classification system based on the metabolic gene expression profile. We performed typing of metabolism-related genes using The Cancer Genome Atlas-Breast Cancer and 2010 (YAU). We included 2,752 metabolic genes reported in previous literature, and the genes were further identified according to statistically significant variance and univariate Cox analyses. These prognostic metabolic genes were used for non-negative matrix factorization (NMF) clustering. Then, we identified characteristic genes in each metabolic subtype using differential analysis. The top 30 characteristic genes in each subtype were selected for signature construction based on statistical parameters. We attempted to identify standard metabolic signatures that could be used for other cohorts for metabolic typing. Subsequently, to demonstrate the effectiveness of the 90 Signature, NTP and NMF dimensional-reduction clustering were used to analyze these results. The reliability of the 90 Signature was verified by comparing the results of the two-dimensionality reduction clusters. Finally, the submap method was used to determine that the C1 metabolic subtype group was sensitive to immunotherapy and more sensitive to the targeted drug sunitinib. This study provides a theoretical basis for diagnosing and treating breast cancer.

## Introduction

Breast cancer is the most common form of cancer in women, and it is the second leading cause of cancer-related deaths in women (Fahad Ullah, 2019). Race, ethnicity, family history of cancer, genetic characteristics, age of menarche, number of pregnancies and births, history of breast biopsies, hormone replacement therapy, alcohol abuse, and physical inactivity are all associated with breast cancer (Budny et al., 2019; Coughlin, 2019). At present, diagnostic methods include breast palpation, ultrasound, molybdenum target, magnetic resonance, and pathological biopsy. The final diagnosis is based on immunohistochemical and cytogenetic tests to accurately assess tumor type, grade, estrogen receptor (ER), progesterone receptor (PR), and human epidermal growth factor receptor 2 (HER2) status (Mueller et al., 2018; Budny et al., 2019). Treatment for breast cancer includes surgery, radiation, and chemotherapy. Patients with early breast cancer, locally advanced breast cancer, and local recurrence can be cured. Nevertheless, many patients have metastases, recurrences, and drug resistance (Carlin Filho et al., 1985).

Cancer classification aids decision-making regarding diagnosis, progression, and prognosis. The traditional classification of breast cancer is based on clinicopathological features and the evaluation of conventional biomarkers. Three broad phenotypes used in clinical practice are ER/PR-positive, HER2-positive, and triple-negative breast cancer (Cadoo et al., 2013). Molecular stratification based on gene expression profiles reveals that breast cancer can be classified as luminal A and B, HER2-enriched, basal-like, and normal-like, and that these subtypes correspond primarily to the status of ER, PR, and HER2 (Russnes et al., 2017). Breast cancer subtypes are determined by morphological, genomic, and proteomic characteristics. The emergence of high-throughput technologies and proteomic innovations facilitates the refinement of breast cancer subtypes (Mueller et al., 2018; Tsang and Tse, 2020). Therefore, it is essential to identify biomarkers and subtypes of breast cancer on a molecular basis to design personalized treatments.

Reprogramming of cell metabolism is a direct and indirect result of carcinogenic mutations and a critical point in cancer therapy (Pavlova and Thompson, 2016). Cancer cells metabolize the nutrients they need to survive and thrive in an environment that often lacks them. Intracellular factors and metabolites regulate the metabolism and behavior of cancer cells in the tumor microenvironment (Vander Heiden and DeBerardinis, 2017; Elia and Haigis, 2021). This study combines the emerging view of metabolic regulation in cancer cells with a biocomputation-based approach to understand metabolism-related phenotypes in breast cancer thoroughly.

## Materials and Methods

### Patients and Samples

We obtained multiple breast datasets from The Cancer Genome Atlas (TCGA, http://cancergenome.nih.gov/) and Genomics of Drug Sensitivity in Cancer (https://www.cancerrxgene.org/). A normalized matrix dataset from multiple studies named Breast Cancer (Yau et al., 2010) was obtained from https://xenabrowser.net. RNA sequencing data (raw counts) of 1,217 and 683 breast carcinoma human samples with detailed clinical information were downloaded from TCGA-BRCA and Breast Cancer (Yau et al., 2010), and the raw counts were transformed into transcripts per kilobase million values for subsequent analysis. TCGA-BRCA and Breast Cancer (Yau et al., 2010) datasets were merged into one metadata set using the SVA R package, which removes batch effects. Then, somatic mutation and copy number data of the BRCA cohort were accessed from the GDAC FireBrowse (http://firebrowse.org/). To investigate drug sensitivity, all solid tumor cell lines with expression level and drug sensitivity data [half-maximal inhibitory concentration (IC_50_) values] were also taken into further analysis (Yang et al., 2013). Finally, we obtained five breast cancer single-cell samples (carcinoma *in situ*) in the GSE180286 (Xu et al., 2021) cohort, GSM5457199, GSM5457202, GSM5457205, GSM5457208, and GSM5457211.

### Breast Carcinoma Metabolism Subclasses

The dataset containing 2,752 metabolism-relevant genes was used to classify the breast carcinoma metabolism subclasses. Non-negative matrix factorization (NMF) clustering distinguished the breast carcinoma metabolism subclasses (Possemato et al., 2011). Before performing NMF on the breast carcinoma samples, we conducted pre-processing analysis on the data. First, candidate genes of low median absolute deviation value (≤0.5) were excluded across all the BRCA patients. The genes obtained were then identified for the second step. The “Survival” package was used for univariate Cox regression analysis with overall survival status as the follow-up endpoint, and the genes with an adjusted *p*-value less than 0.05 were identified and applied to the subsequent analysis. We obtained metabolic genes that were high-variable (median absolute deviation >0.5) and significantly associated with outcome. Then, the NMF R package was applied to the NMF clustering of the resulting matrix, and several breast cancer subgroups with different metabolic characteristics were obtained. This method was also applied to Breast Cancer (Yau 2010) using the same candidate genes.

### Gene Set Variation Analysis (GSVA)

GSVA is a nonparametric and unsupervised gene set enrichment method that evaluates each breast carcinoma sample’s metabolism signature score (Hänzelmann et al., 2013). Each sample was given 115 metabolism signatures scores by the GSVA R package (Désert et al., 2017; Rosario et al., 2018).

Subsequently, metabolism signature differential analysis was applied based on GSVA results. The signatures with a log2 fold change (FC) > 0.2 (adjusted *p* < 0.05) were selected as differentially-expressed signatures and used for further study.

### Estimation of Immune Infiltration

Microenvironment cell population-counter (MCP-counter) methods were used to assess the proportion of immune cell abundance in breast cancer tissue, Published by FEBS Press and John Wiley & Sons Ltd. We also used single-sample GSEA (ssGSEA), which calculates each dataset’s scores for all samples (Barbie et al., 2009). The datasets included regulatory T cells, T helper cell 1, T helper cell 2, T helper cell 17, central memory T cell, and effective memory T cell. The ESTIMATE algorithm was used to evaluate immune scores and stromal scores related to the tumor microenvironment (Yoshihara et al., 2013).

### Characterization of Breast Cancer Subclasses

The “Limma” package was used to determine differentially-expressed genes (DEGs) among breast carcinoma subclasses with log2 FC > 1 (adjusted *p* < 0.01). We downloaded “c2.cp.kegg.v6.2.symbols” and “h.all.v6.2.symbols” from the Molecular Signatures Database. The biological function for DEGs was analyzed using the CLUSTERPROFILER R package (Yu et al., 2012). The biological function terms with adjusted *p* < 0.05 were considered significant.

### Generation of the Classifier and Performance Validation

The statistically significant DEGs were defined as adjusted *p* < 0.01 and |log2 FC > 2| in each subgroup. We identified the top 30 characteristic genes with the maximum |log2FC| value in each group (only the genes with log2FC > 0 were selected) and constructed the prediction model of breast cancer metabolic typing using 90 characteristic genes in the three groups.

### Prediction of the Benefit From Immunotherapy and Targeted Therapy for Each Subclass

The immunotherapies cohort was used to verify the correlation between metabolism subclasses and immune checkpoint therapy efficacy. The metabolism subclasses were determined using the same we used in TCGA-BRCA based on submap analysis (Gene Pattern) (Roh et al., 2017). Subsequently, drug susceptibility was performed to analyze the various drug responses in the three subclasses. Drug-sensitive cell lines were defined as the top 1/3 of IC_50_ values, and drug-resistant cell lines were defined as the bottom 1/3 of IC_50_ values.

### Single-Cell Data Pre-process

The raw gene matrix for GSE180286 was obtained from the GEO database, Seurat package was applied to process data in R (Stuart et al., 2019). The raw data GSE180286 were loaded with Seurat, and cells were filtered with the criteria of >20% mitochondria-related genes or more than 6,000 genes expressed. Merged cells data were clustered into ten cell populations using FindClusters (resolution = 0.3). Meanwhile, UMAP reduction of cell clustering was performed. We used function SCTransform in Seurat to perform data normalization and used CCA to de-batch five samples of breast cancer original tumor after we separated ten cell subtypes.

### scMetabolism

We used Harmony (Korsunsky et al., 2019) method to de-batch epithelial cells. SCmetabolism (Wu et al., 2021) was used to assess the activation of metabolic pathways in epithelial cells. We used VISION’s approach to assess metabolic activation of different subtypes of epithelial cells. Subgroups were used to evaluate the relationship between epithelial cell subtypes and metabolic subtypes in TCGA.

### Statistical Analysis

The unpaired Student’s *t*-test was used to compare groups with normally distributed variables, and the Mann–Whitney *U*-test was used to compare groups with non-normally distributed variables. One-way analysis and Kruskal–Wallis tests of variance were used as parametric and nonparametric methods to compare three groups, respectively. Contingency table variables were analyzed using the chi-square test or Fisher’s exact tests. Survival analysis was performed using Kaplan-Meier methods and compared using the log-rank test. A univariate Cox proportional hazards regression model was used to estimate the hazard ratios for univariate analyses. All statistical analyses were performed using R 3.6.3 (https://www.r-project.org/).

## Results

### The Metabolic Subtypes of Breast Cancer

**FIGURE 1:**
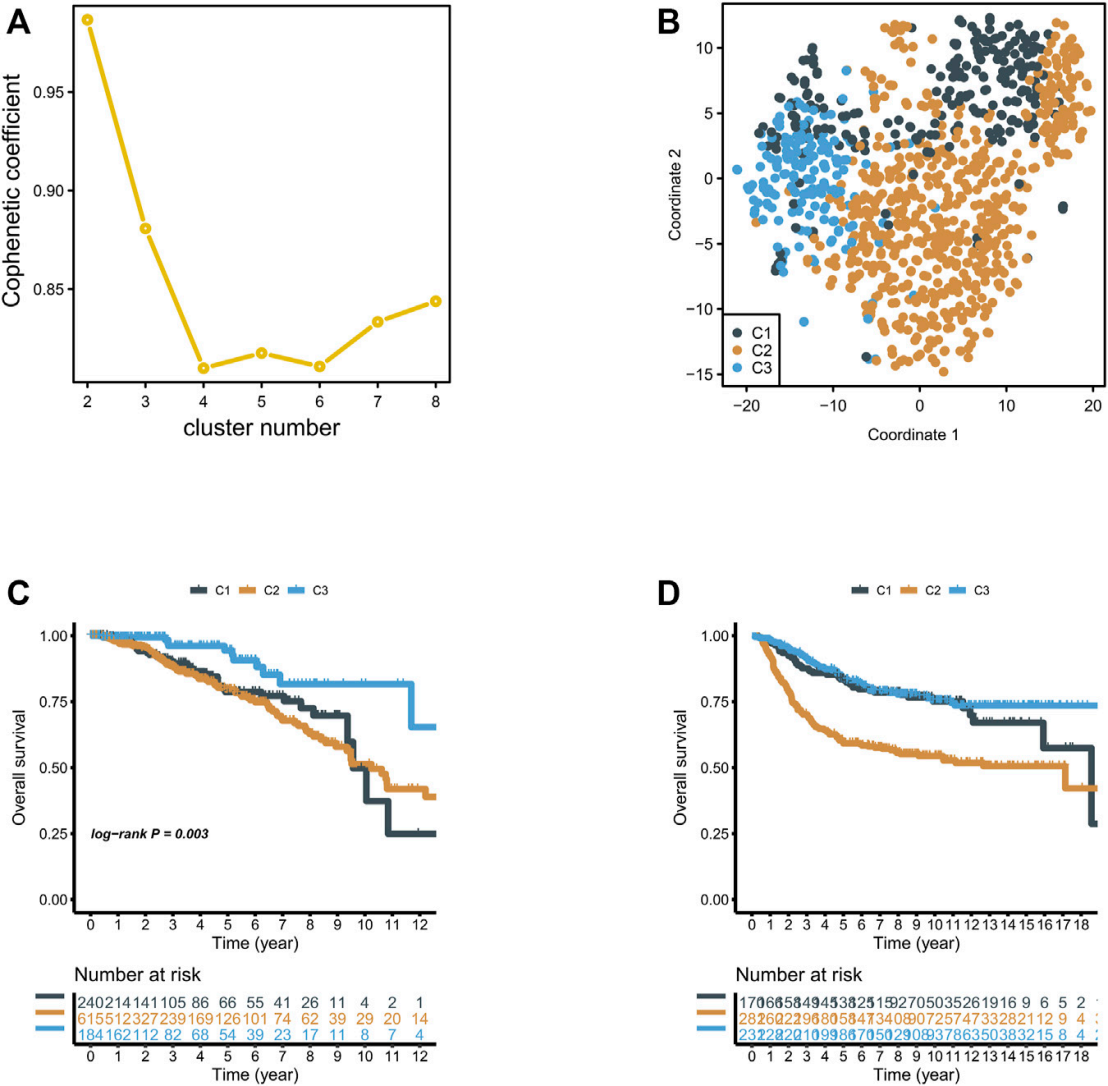
**(A)** NMF clustering was performed for 816 metabolism-related genes, and the affinity correlation coefficient was *k* = 2–8; **(B)** Classification of breast cancer into three subgroups using T-SNE analysis; **(C,D)** Overall survival of three subgroups in the TCGA and Xena Breast Cancer (Yau 2010) cohorts.

We included previously reported 2,752 metabolism-related genes (Supplementary Table S1). First, metabolism-related genes with a variance of zero were excluded. Then, univariate Cox regression analysis was applied, and 117 metabolism-related genes were identified according to the corrected *p*-value of regression analysis less than 0.01 (Supplementary Table S2). We then grouped 117 metabolism-related gene matrixes according to non-negative matrix classification. Cophenetic correlation coefficients were applied to identify the optimal *p*-value, and *K* = 3 was finally determined as the optimal clustering result (Figure 1A). To verify the degree of classification before the three metabolic groups we obtained, we used TSNE dimension reduction. We found that clustering dispersion existed among metabolic subgroups (Figure 1B). According to the subtypes of the various metabolic groups, we calculated overall survival and found that there were significant differences in survival in groups C1, C2, and C3, among which the C2 group had poor outcomes in TCGA and YAU2010 cohort, while the C3 group had a good outcome (Figures 1C,D).

### Metabolism Analysis Among Different Subclasses

**FIGURE 2:**
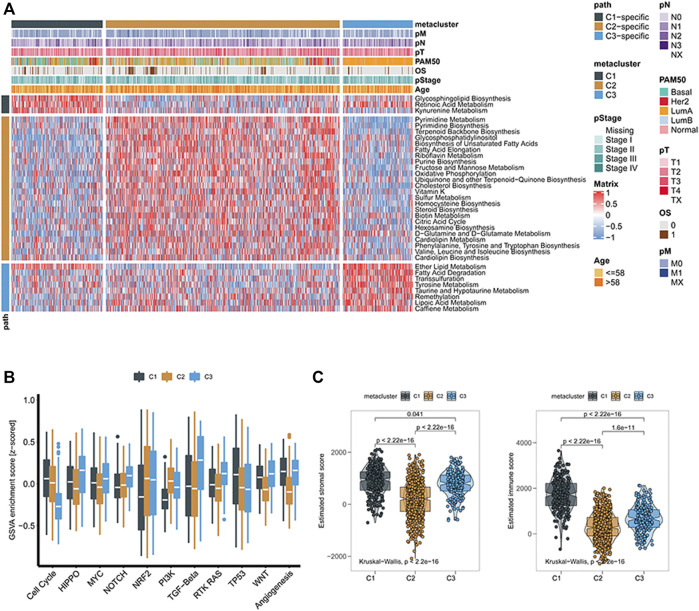
**(A)** Heatmaps of breast cancer gene signatures associated with metabolism; **(B)** Boxplots of characteristic scores associated with cancer progression for different subclasses of breast cancer; **(C)** Box plots of stroma and immune scores of tumor microenvironment in different subclasses of breast cancer.

We obtained three metabolic groups with different prognostic values based on the metabolic gene classification. To explore the metabolic characteristics of the three groups, we identified the characteristic metabolic pathways of the three subtypes. First, we used the GSVA method to evaluate 113 metabolic processes in breast cancer (Supplementary Table S3). We then analyzed the differences of 115 metabolic processes in the three groups. The characteristic metabolic pathways of C1 were retinoic acid metabolism and kynurenine metabolism, and the characteristic metabolic pathways of C2 were pyrimidine metabolism, riboflavin metabolism, mannose metabolism, and sulfur metabolism. The characteristic metabolic pathway was ether lipid metabolism, tyrosine metabolism, and lipoic acid metabolism (Figure 2A).

To further explore the biological processes of the subgroups, we used GSVA analysis to determine that there were also significant differences in cell cycle, HIPPO, MYC, NOTCH, NRF2, PI3K, TGF-Beta, RTK RAS, TP53, Wnt, and angiogenesis. The C1 and C3 groups had higher Wnt pathway scores, suggesting that C1 and C3 groups were more closely related to *β*-catenin-related proteins (Figure 2B). We then found that the immune score and matrix score of the three metabolic groups were significantly different, among which the immune score of the C1 group was higher (Figure 2C).

### Determine the Immunological Panorama of Different Metabolic Subtypes

**FIGURE 3:**
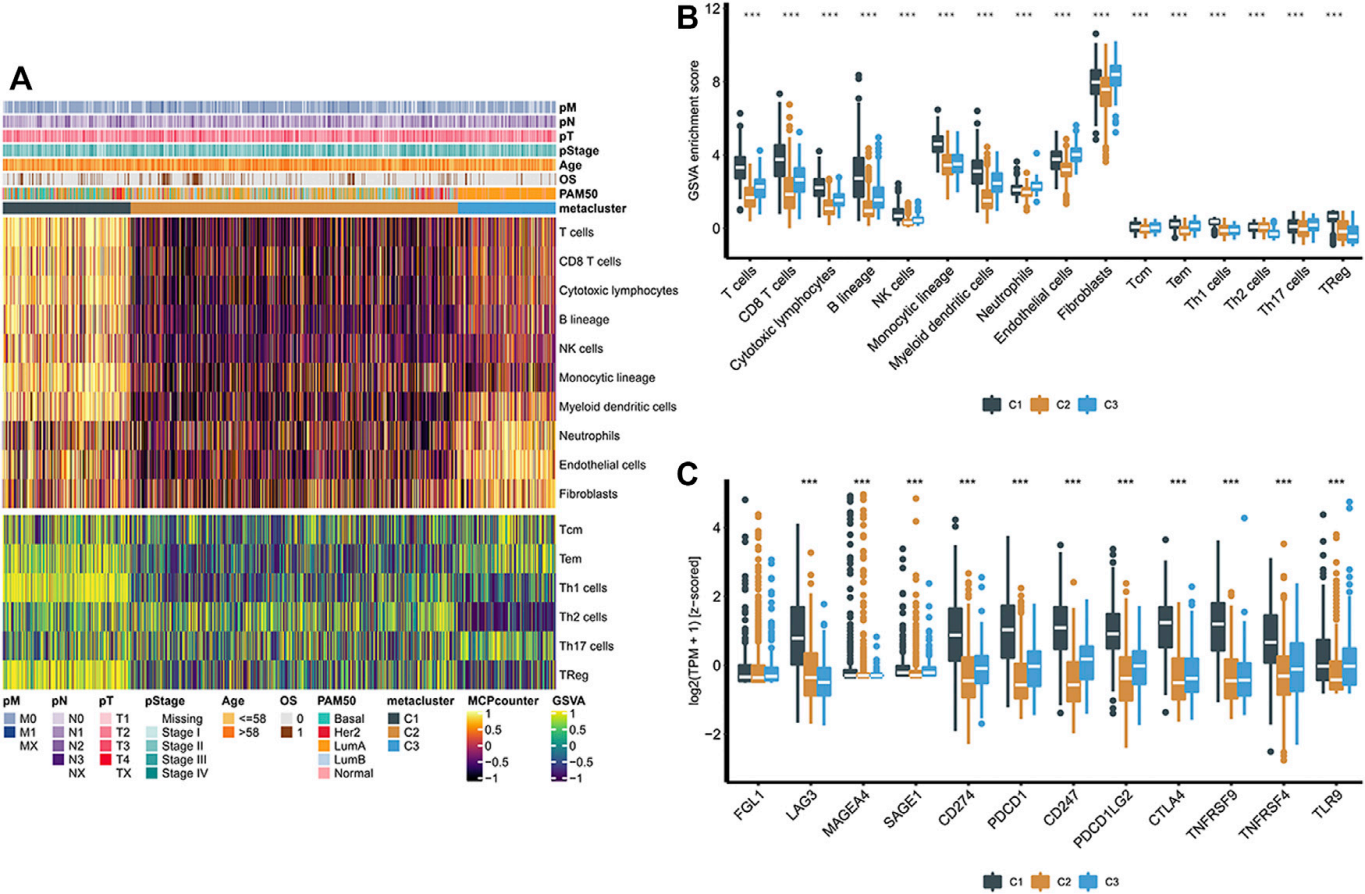
**(A)** Heatmaps of the population abundance of immune cells and stromal cells in the three subtypes of breast cancer; **(B)** Enrichment scores of different immune cells and stromal cells in three subclasses of breast cancer; **(C)** Boxplot showing the expression levels of 12 immune checkpoint genes in three breast cancer subclasses. **** indicates that *p* < 0.0001.

We used MCP-counter and the ssGSEA algorithm to calculate the relative abundance of 16 types of immune cells. The abundance of immune cells corresponding to the three groups evaluated by the MCP-counter algorithm is shown in Figure 3A. C2 was significantly different from the other two subgroups, and the abundances of ten types of immune cells in the C2 subgroup were low, including T cells, CD8^+^ T cells, cytotoxic lymphocytes, the B lineage, NK cells, the monocytic lineage, myeloid dendritic cells, neutrophils, endothelial cells, and fibroblasts (Figure 3A). It is noteworthy that consistent results were obtained in the GSVA method (Figure 3B). Later, we investigated the association between three metabolic subtypes and the expression levels of 12 immune checkpoint treatment-related biomarkers. Immune checkpoint genes were chosen based on current clinical trials or drug inhibitors demonstrated to be effective. The expression levels of nine immune checkpoint genes in the C1 group were significantly increased, suggesting that the C1 group would show better therapeutic efficacy of immune checkpoint inhibitors (Figure 3C).

### Correlation of the Breast Cancer Subclasses With Multi-Omics

**FIGURE 4:**
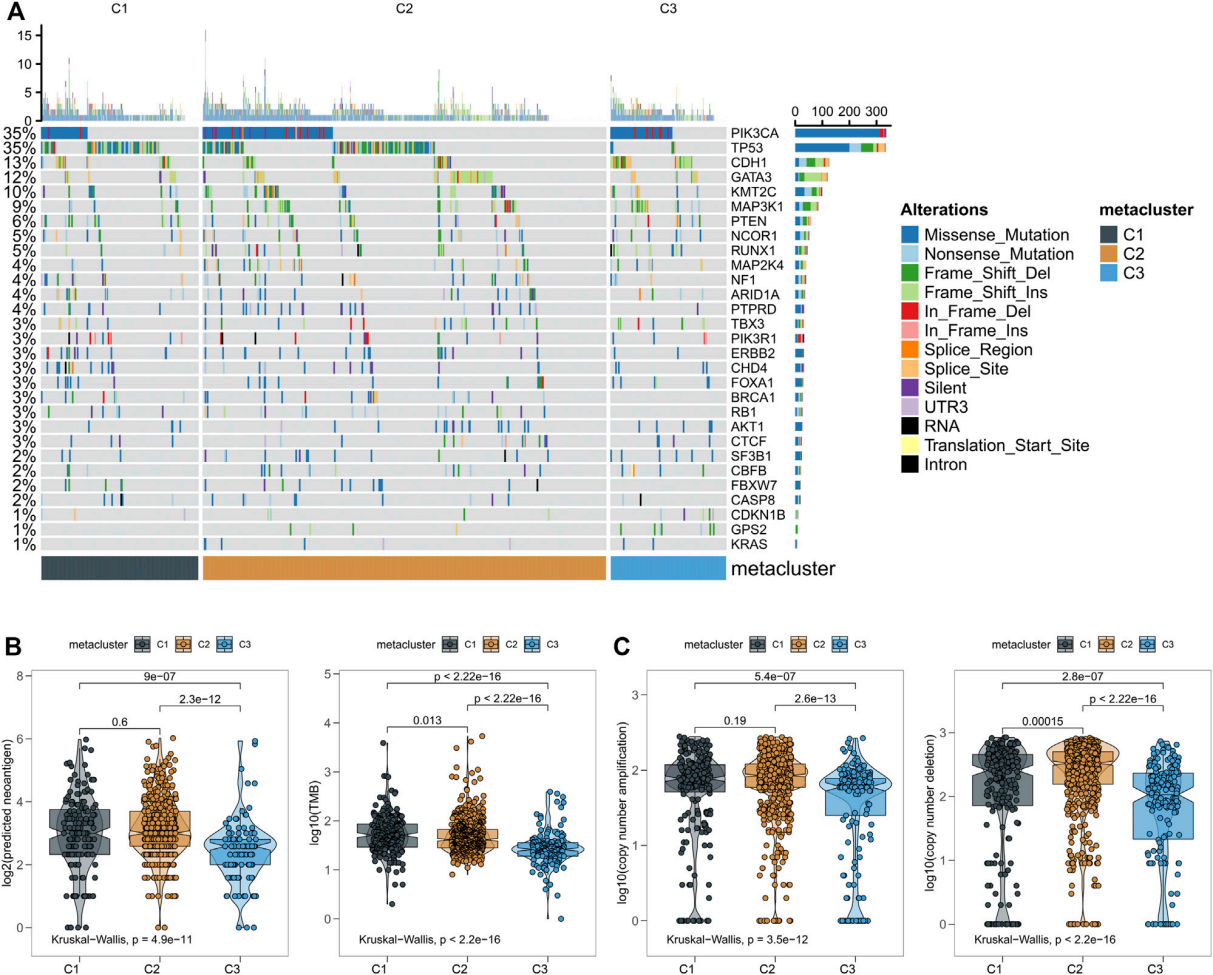
**(A)** The relationship between three subtypes of breast cancer and the classical pathway of mutation and related genes; **(B)** Number of mutations and predicted neoantigens in the three breast cancer subgroups; **(C)** Copy number aberrations in three breast cancer subtypes.

According to this analysis, anti-tumor immunity differs across metabolic groups. To explore the differences in somatic mutation frequency and mutation mode in these groups, we obtained somatic mutation data in breast cancer from TCGA. The genes with high mutation frequency are shown in Figure 4A. C3 displayed distinct mutation characteristics. Specifically, the mutation frequency of PIK3CA in C3 was significantly higher than C1 and C2. Subsequently, we found significant differences in new tumor antigen, tumor mutation burden, copy number amplification, and copy number reduction in the three metabolic groups (Figures 4B,C). These findings suggest significant differences in expression and regulation patterns among the three metabolic subtypes.

### 90—Metabolic Gene Signature

**FIGURE 5:**
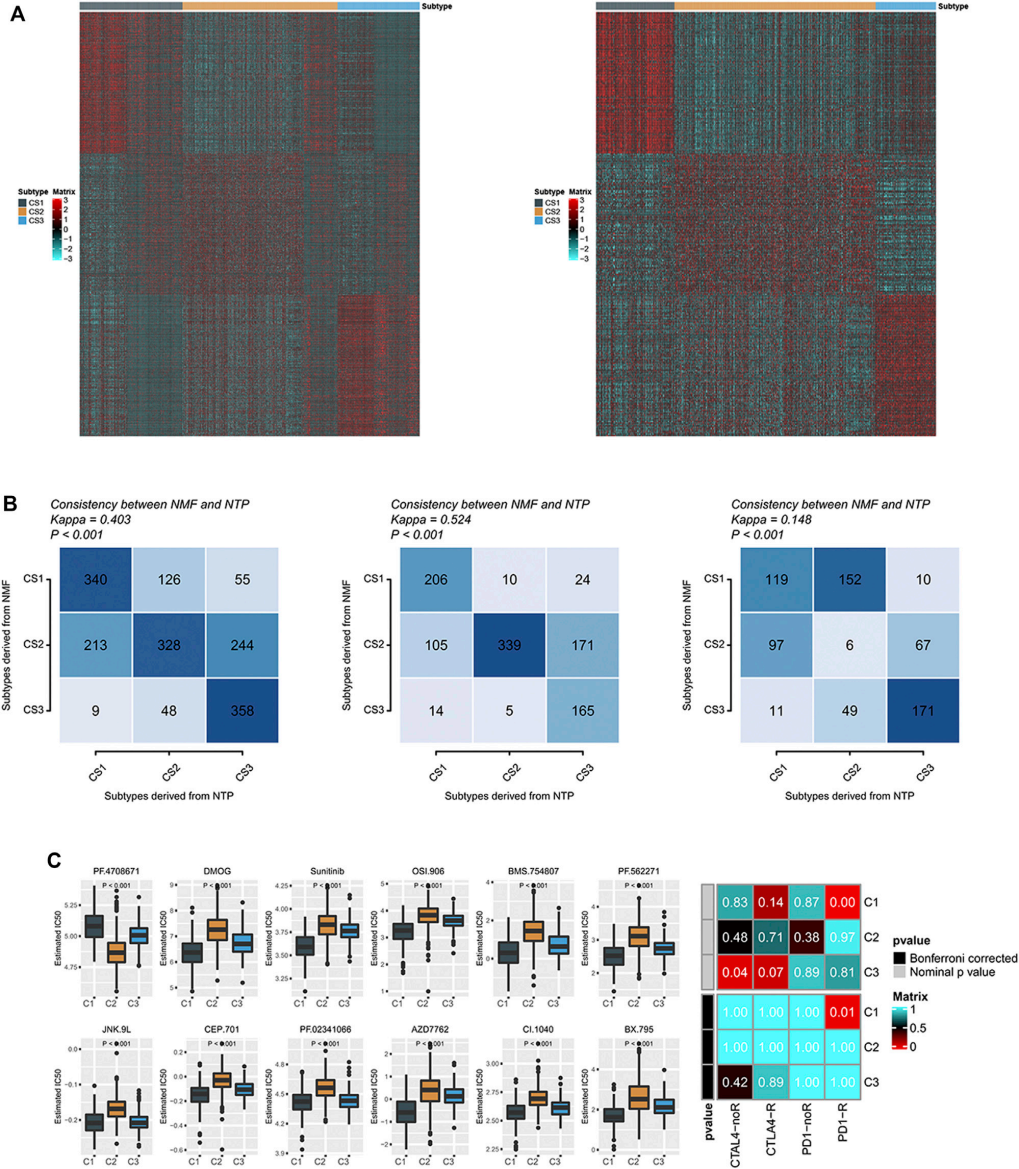
**(A)** Heatmaps of the expression levels of 90 gene classifiers; **(B)** The consistency between the prediction of molecular subclasses of breast cancer by 90 gene classifiers and the original prediction based on NMF; **(C)** There were significant differences in sensitivity analysis of 12 drugs among different subtypes of breast cancer. Sensitivity of different subclasses of CTLA-4 inhibitors and PD-1 inhibitors in breast cancer.

A non-negative matrix classification was carried out based on prognostic metabolic factors, and three metabolic subtypes were obtained. To apply this classification method to other breast cancer cohorts, we constructed a gene set that could be used for non-negative matrix classification. First, we performed a differential analysis of C1 and non-C1 cohorts in the breast cancer cohort, obtaining the top 30 genes by significance sequencing, and the same principle was applied to the C2 and C3 cohorts. These 90 genes are characteristic and can be used for metabolic classification of other cohorts. The upregulated gene expression profile of each group in TCGA-BRCA is shown on the left side of Figure 5A, and the same profile of the combined cohort of TCGA-BRCA and Breast Cancer (Yau 2010) is shown on the right side. Subsequently, we matched non-negative matrix results of 90 feature gene sets using NTP classification results in the combined cohort, TCGA-BRCA, and Breast Cancer (Yau 2010) cohorts. We found that the results of the two classification strategies were similar (Figure 5B). These findings suggest that the 90-gene signature could be extended to other cohorts for metabolic typing.

### Sensitivity Analysis of Different Metabolic Subtypes of Breast Cancer to Immunotherapy

We identified differences in the immune microenvironment and immune checkpoint levels in various metabolic groups of breast cancer, suggesting that the three groups have varying sensitivities to immune checkpoint treatment. There was a strong correlation between the C1 group and immune checkpoint inhibitor gene expression level, possibly suggesting that the C1 group would respond favorably to immune checkpoint treatment. To test this inference, we compared the expression patterns of C1, C2, and C3 groups in breast cancer with the expression patterns of patients with different response outcomes in the immune checkpoint treatment cohort using a submap. We found that the expression profile of the C1 group in TCGA significantly correlated with the PD-1 checkpoint inhibitor response group (*p* = 0.01), suggesting that the C1 group was more likely to respond to PD-1 therapy (Figure 5C). We also measured the sensitivity of targeted drugs in different patients (Figure 5C). As a targeted drug for breast cancer treatment, Sunitinib had a lower IC_50_ in group C1, suggesting that patients in group C1 were also more susceptible to targeted drugs (Figure 5C).

### Single-Cell Analysis

**FIGURE 6:**
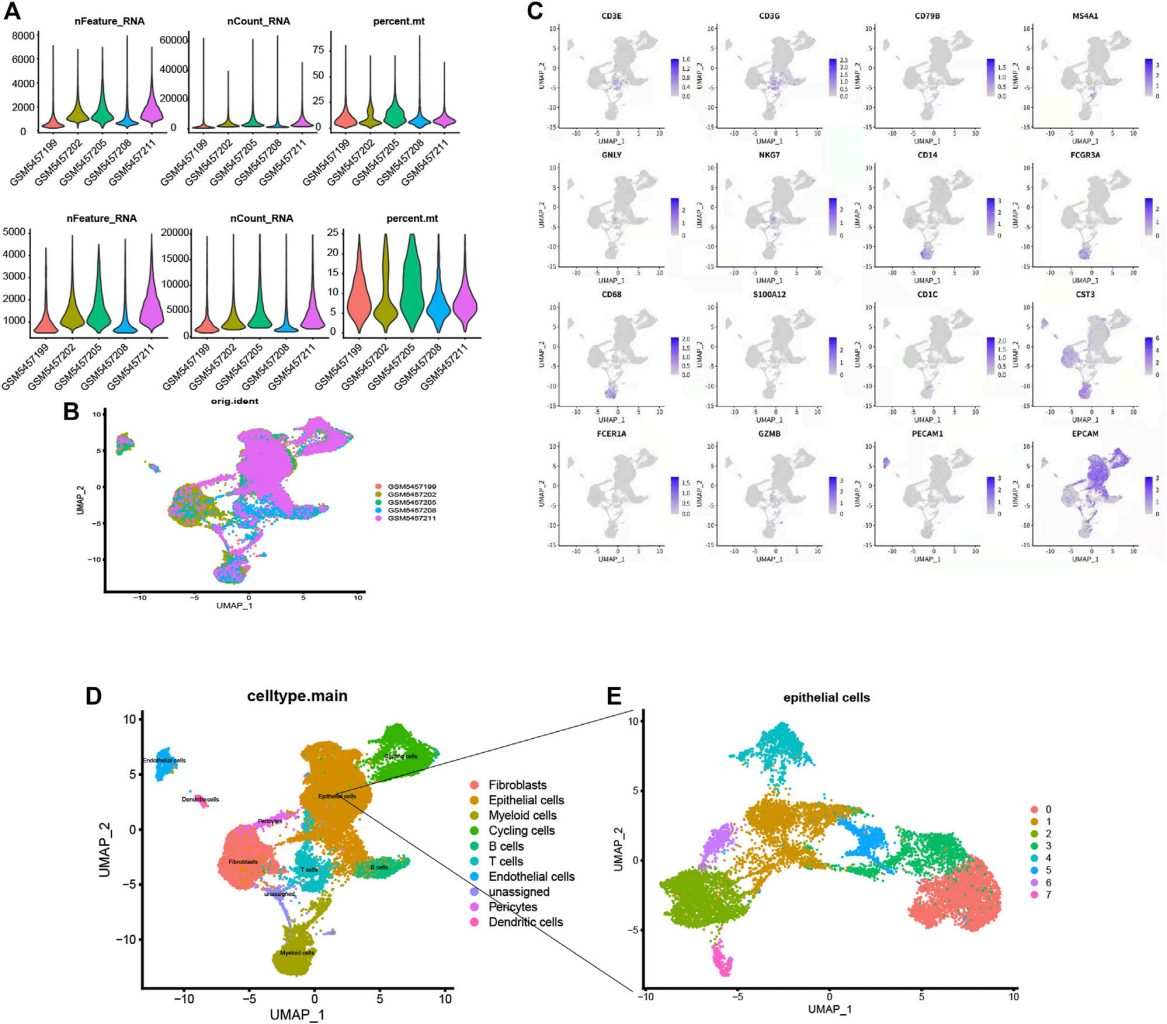
**(A)** The data pre-process results. **(B)**. The distribution of the five samples. **(C)** Fibroblasts, Epithelial cells, Myeloid cells, Cycling cells, B cells, T cells, Endothelial cells, unassigned, Pericytes, and Dendritic cells. For example, CD3E, CD3E, CD79B and MS4A1 **(D)** Subgroup annotation analysis for single cells, **(E)** Eight types of epithelial subgroups.

GSM5457199, GSM5457202, GSM5457205, GSM5457208, and GSM5457211 were taken into this study. The data pre-process results are shown in Figure 6A. Based on the UMAP dimension reduction clustering, we obtained ten cell clusters. They were Fibroblasts, Epithelial cells, Myeloid cells, Cycling cells, B cells, T cells, Endothelial cells, unassigned, Pericytes, and Dendritic cells. The markers of these cells were shown in Figure 6B, for example, CD3E, CD3E, CD79B, and MS4A1. The cluster results of the five samples are shown in Figure 6C. Afterward, we performed subgroup analysis for epithelial cells in Figure 6D, and we obtained eight epithelial subgroups (Figure 6E).

### Determine Characteristic Metabolism Pathway in the Single R Cohort

**FIGURE 7:**
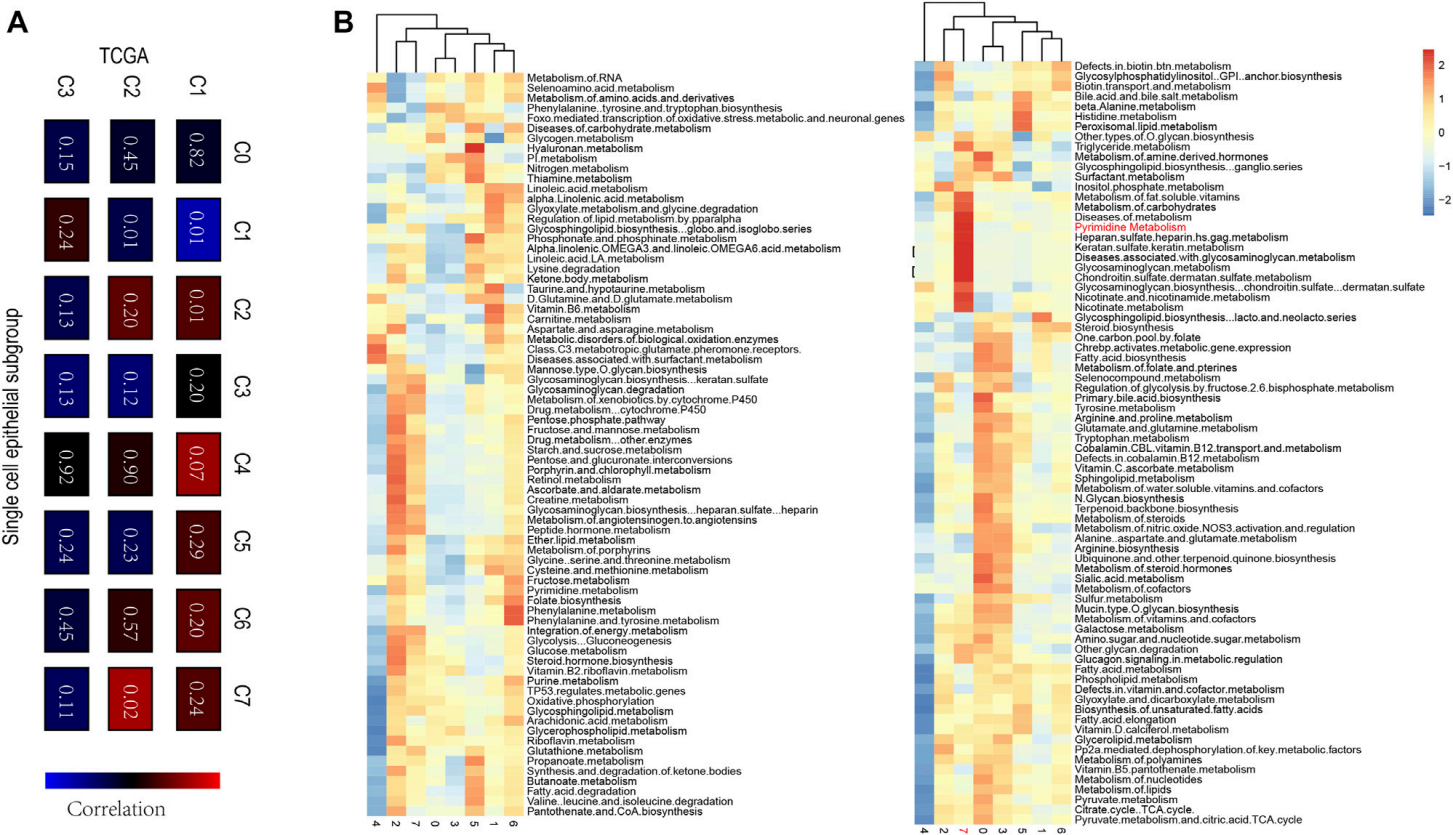
**(A)** The correlation among TCGA c1-C3 and single-cell C0-C7, a significant positive correlation between the C2 group in the TCGA subtype and the C7 group in the breast cancer epithelial cell group were evaluated (*p* = 0.02) **(B)** The characteristic metabolic pathways in epithelial subgroups determined by SCmetabolism, and Pyrimidine Metabolism pathway could be used as a characteristic metabolic pathway of breast cancer tumor heterogeneity.

To demonstrate the heterogeneity of metabolic pathways in TCGA, we matched the subtypes obtained in the TCGA breast cancer cohort with eight epithelial subtypes of single cells. We found a significant positive correlation between the C2 group in the TCGA subtype and the C7 group in the breast cancer epithelial cell group (*p* = 0.02) (Figure 7A). To further explore the common characteristic metabolic pathways in C2 and C7 groups, SCmetabolism was used to calculate and evaluate the characteristic metabolic pathways in epithelial subgroups. We found that pyrimidine metabolism was significantly upregulated in TCGA C2 and single-cell C7 groups (Figure 7B). Therefore, we believe that pyrimidine metabolism pathway A can be used as a characteristic metabolic pathway of breast cancer tumor heterogeneity.

## Discussion

The subclassification of breast cancer has no unified conceptual framework in molecular taxonomy. In the present study, three breast cancer subtypes were identified based on identified metabolic genes, and we explored their metabolic characteristics, clinical characteristics, tumor immune microenvironment, mutation load, drug sensitivity, and other aspects.

Among the three subtypes of breast cancer, the survival outcome of C3 was better than that of C1 and C2. The primary metabolic pathways of C3 enrichment include fatty acid degradation and metabolism of ether lipids, transsulfuration tyrosine, taurine and hypotaurine, remethylation lipoic acid, and caffeine. Better survival outcomes for C3 may benefit from this enriched metabolic pattern. Abnormal lipid metabolism is a hallmark of cancer cells, and changes in lipid metabolism affect growth processes in cancer cells. Sphingolipids and non-sphingolipids were strongly increased in breast cancer cells. The expression level of ether lipids was significantly higher in breast cancer cells than normal cells (Hahnefeld et al., 2020). Ether lipid levels are elevated in tumors; in several invasive cancer cells and primary tumors, alkyl glycerophosphate synthase controls the utilization and metabolism of cytoether phospholipids to promote cancer invasion and tumor growth (Benjamin et al., 2013; Piano et al., 2015; Stazi et al., 2019).

Iron death is a non-apoptotic cell death process associated with targeted susceptibility to certain cancers. Lipomic analysis was also used to find that polyunsaturated ether phospholipids act as substrates for lipid peroxidation to induce iron death (Zou et al., 2020). In lipid metabolism, the synthesis of fatty acids is vital for cancer cell proliferation. Fatty acids convert nutrients for membrane biosynthesis, energy storage, and signal molecule production (Röhrig and Schulze, 2016). Thus, limiting the supply of fatty acids can limit the proliferation of cancer cells, including by increasing the degradation of fatty acids through oxidation (Koundouros and Poulogiannis, 2020). C3 is related to several well-known oncogenic signaling pathways, including HIPPO, TGFB, RTKRAS, and angiogenesis, and is affected by several pathways related to abnormal tumor metabolism (Chiang et al., 2016; Viallard and Larrivée, 2017; Panciera et al., 2020; Park et al., 2020). In normal cells, the primary function of the MYC pathway is to coordinate nutrient acquisition energy, triggering selective gene expression amplification to promote cell growth and proliferation. In cancer, genetic and epigenetic disorders deregulated transcription factors in the MYC family, and carcinogenic levels of MYC reprogram cell metabolism to promote the growth and proliferation of cancer cells, a hallmark of cancer development (Dejure and Eilers, 2017). Uncontrolled growth in response to misregulated MYC expression depends on MYC-driven metabolic pathways (Stine et al., 2015). MYC directly regulates glycolytic genes to regulate glucose metabolism and indirectly regulates gene expression to increase glutamine metabolism. Ectopic MYC expression in cancer drives aerobic glycolysis and oxidative phosphorylation (Dang et al., 2009).

The NOTCH signaling pathway is involved in angiogenesis, tumor immunity, and drug resistance and plays a role in tumor metabolism (Majumder et al., 2021). In tumors with highly acquired NOTCH mutations, MYC is a common target gene that drives NOTCH-dependent tumor cell growth and metabolism (Aster et al., 2017). The Wnt pathway is upregulated in most breast cancer patients and is associated with poor outcomes. The Wnt signaling pathway is involved in the proliferation and metastasis of breast cancer and participates in immune microenvironment regulation, drug resistance, and phenotypic shaping of breast cancer (Xu et al., 2020a). Active Wnt signaling also participates in cancer cell metabolic reprogramming. Through C-MYC, Wnt controls glutamine transport and production. The Wnt pathway partially regulates pyruvate carboxylase and pyruvate dehydrogenase kinases and upregulates aerobic glycolysis through Wnt-mediated transcriptional changes in *β*-catenin (Sherwood, 2015; El-Sahli et al., 2019).

The C2 subclass is closely associated with several metabolic pathways. Purine and pyrimidine are the key molecules in cellular biological processes such as DNA replication and RNA synthesis. Pyrimidine metabolism is one of the primary pathways of significant enrichment and dysregulation of transcription levels in many cancers (Edwards et al., 2016; Siddiqui and Ceppi, 2020). Pyrimidine antineoplastic agents such as gemcitabine and cytarabine and purine antineoplastic agents such as 6-thioguanine and 6-mercaptopurine are commonly used to treat cancers (Fridley et al., 2011). As antimetabolites, these drugs compete with physiologic pyrimidine and purine nucleosides and interact with many intracellular targets to induce cytotoxicity to kill cancer cells (Galmarini et al., 2003). Some anticancer drugs have been used to inhibit the biological function of mitochondria and directly inhibit respiratory chain complexes (Sancho et al., 2016; Sica et al., 2020). In contrast to normal cells, which rely primarily on mitochondrial oxidative phosphorylation for energy production, most cancer cells rely on aerobic glycolysis to produce ATP, known as the Warburg effect (Vander Heiden et al., 2009). However, recent evidence suggests that oxidative phosphorylation (OXPHOS) also plays a critical role in cancer progression. Moreover, increased OXPHOS dependence is generally characteristic of cancer stem cells. Cancer cells can enter a state of coexistence of oxidation and glycolysis (Yu et al., 2017; Ashton et al., 2018).

Cholesterol metabolism produces membrane components necessary for cells and produces a variety of metabolites with biological functions (Kopecka et al., 2020). In the tumor microenvironment, cholesterol metabolism is reprogrammed to promote tumorigenesis (Huang et al., 2020). Blocking cholesterol synthesis and uptake inhibits tumor development (Xu et al., 2020b). Steroid hormones and their precursors are synthesized and extensively metabolized in the adrenal glands and gonads (Capper et al., 2016). Breast cancer is hormone-dependent, and cancerous breast tissue promotes mitosis by expressing ER, AR, and PR receptors. The local estrogen biosynthesis is believed to play an indispensable role in developing hormone-dependent breast cancer (Foster, 2008).

The tricarboxylic acid (TCA) cycle is an essential metabolic pathway for generating energy supporting life activities. It stimulates increased glutamine breakdown, glycolysis and produces reactive oxygen species. Metabolites derived from the TCA cycle mediate signal transduction in immune cells (Scagliola et al., 2020). Furthermore, genetic changes in the TCA cycle enzymes lead to the production of tumor metabolic intermediates, suggesting that changes in mitochondrial metabolism are an essential driving force of cancer initiation and progression (Sajnani et al., 2017).

Cardiolipin (CL) is a specific phospholipid of mitochondria, and dysregulation of CL metabolism has been observed in several types of cancer (Ahmadpour et al., 2020). In cancer cachexia, increased CL content may induce higher energy consumption of mitochondria (Peyta et al., 2015). C2 is regulated by the familiar oncogenic pathways, NRF2 and PI3K (He et al., 2020). NRF2 regulates oxidative stress and growth factor signaling. Nutritional status and NRF2-mediated metabolic dysregulation support cancer cell proliferation (Lee et al., 2018). The PI3K-AKT-MTOR signal transduction pathway regulates various biological processes, including cell growth and proliferation, cell cycle progression, cell metabolism, and cytoskeletal reorganization (Papa and Pandolfi, 2019). The PI3K-Akt-mTOR pathway is a central regulator of glycolysis that promotes cancer metabolism and proliferation (Courtnay et al., 2015).

The immune pathway enrichment and immune infiltration scores in C1 subtype breast cancer were higher than in C2 and C3. In drug sensitivity analysis, C1 responded significantly to immune checkpoint inhibitors. We hypothesized that the C1 subgroup of breast cancer patients might be most responsive to immunotherapy with immune checkpoint inhibitors. Subtype C1 has also been implicated in cancer metabolism. MYC increases intracellular levels of tryptophan and tryptophan metabolites in the canine uridine metabolic pathway, thereby meeting the needs of the rapid proliferation of cancer cells (Venkateswaran et al., 2019). Tumor reproliferating cells drive PD-1 upregulation in CD8 + T cells through the canine urine-aromatics receptor pathway, which may be a potential mechanism for tumor immunotherapy (Liu et al., 2018). Retinoic acid is the primary bioactive metabolite of retinol (vitamin A). The destruction of RA is the basis of the development of many malignant tumors (di Masi et al., 2015). Retinoids and their natural metabolites and synthetic products induce the differentiation of several cell types. Among them, fenvitamine may have a preventive effect in young women at high risk for breast cancer (Connolly et al., 2013).

Similar to genetic heterogeneity, metabolic phenotypes in cancer are highly heterogeneous. This study explored the metabolic landscape of breast cancer and identified three subgroups of breast cancer with different metabolic activities. This new classification assists breast cancer diagnosis, treatment, and outcome.

## Data Availability

The datasets presented in this study can be found in online repositories. The names of the repository/repositories and accession number(s) can be found in the article/Supplementary Material.

## Supplementary Material

Supplementary Table S1 2752 metabolism-related genes list.

Supplementary Table S2 Univariate Cox regression analysis was applied, and 117 metabolism-related genes were identified according to the corrected P-value of regression analysis less than 0.01.

Supplementary Table S3 The GSVA result of 113 metabolic processes in breast cancer
